# Surface active properties of lipid nanocapsules

**DOI:** 10.1371/journal.pone.0179211

**Published:** 2017-08-10

**Authors:** Celia R. A. Mouzouvi, Anita Umerska, André K. Bigot, Patrick Saulnier

**Affiliations:** 1 ‘Micro et Nanomédecines biomimétiques—MINT‘, INSERM U1066 Université d’Angers, CNRS 6021, Université Bretagne Loire, Angers, France; 2 Laboratoire de Pharmacie Galenique et de technologie Pharmaceutique, UFR Pharmacie, FSS, Université d’Abomey-calavi, Cotonou, Benin; 3 Unité d’Immunologie, Faculté des Sciences de la Santé, Université d’Abomey-calavi, Cotonou, Benin; Universidade Estadual Paulista Julio de Mesquita Filho, BRAZIL

## Abstract

Lipid nanocapsules (LNCs) are biomimetic nanocarriers used for the encapsulation of a broad variety of active ingredients. Similar to surface active compounds, LNCs contain both hydrophilic and hydrophobic parts in their structure. Moreover, the components of LNCs, macrogol 15 hydroxystearate (MHS) and lecithin, are known for their surface active properties. Therefore, the aim of this paper was to investigate the capability of the LNCs to decrease surface tension using two techniques: drop tensiometry and the Wilhelmy plate method. LNCs with diameters ranging from 30 to 100 nm were successfully obtained using a phase inversion technique. The LNCs’ properties, such as size and zeta potential, depend on the composition. LNCs exhibit a lower limiting surface tension compared to MHS (34.8–35.0 mN/m and 37.7–38.8 mN/m, respectively), as confirmed by both drop tensiometry and the Wilhelmy plate method. LNCs have exhibited a saturated interfacial concentration (SIC) that was 10-fold higher than the critical micellar concentration (CMC) of MHS or the SIC of binary and ternary mixtures of LNC ingredients. The SIC of the LNC formulations depended on the mass mixing ratio of the MHS/triglycerides but not on the presence of lecithin. The CMC/SIC values measured by the Wilhelmy plate method were higher than those obtained using drop tensiometry because of the longer duration of the tensiometry measurement. In conclusion, the surfactant-like properties of the LNCs offer new possibilities for medical and pharmaceutical applications.

## Introduction

Lipid nanocapsules (LNCs) are biomimetic nanocarriers with a structure that is a hybrid between polymeric nanoparticles and liposomes. LNCs contain an oily core composed of medium chain triglycerides surrounded by a surfactant shell made of a PEGylated surfactant and optionally lecithin or other co-surfactants [1,2]. All above-mentioned LNC components are approved by the FDA for oral, topical and parenteral administration [1]. Numerous active ingredients, mainly drugs with lipophilic properties, have already been incorporated into LNCs, including fluticasone propionate [3], essential oils: eugenol, carvacrol and trans-cinnamaldehyde [4], paclitaxel [5] and ibuprofen [6]. Recently, interest in the use LNCs as carriers for hydrophilic compounds (e.g., polymyxin B, calcitonin and antimicrobial peptides) using adsorption processes has been developed [2]. LNCs are carriers for various administration routes, including pulmonary [3,5], intravenous [7], oral and local delivery [8]. The advantages of LNCs include small particle size (20–100 nm), good physical stability (18 months) and manufacturability via a phase inversion temperature method, which is a low energy, organic solvent-free process [8,9]. Physical properties, such as particle size and zeta potential, and biological properties of the LNCs have extensively been studied since 2002 [3,8,10–13]. Although there have been reports on other LNC physical properties, such as elasticity [13], no study on the ability of LNCs to decrease surface tension has been performed to date.

Surface tension is the elastic tendency of a fluid surface to acquire a minimum surface area. At a molecular level, this phenomenon results from the difference in energy between molecules at a fluid interface and in the bulk [14]. The interfacial/surface tension is fundamentally important to colloid science [14]. Advancements in surface chemistry have led to countless applications in several industries. For instance, interfaces are critically important in pharmaceutics, biotechnology and biomedicine [15]. In pharmaceutical sciences, interfacial phenomena play an important role in the processing of a variety of formulations. The interactions between particles and interfaces may occur during the processing (e.g., spray drying) or administration (e.g., nebulization). The subsequent behaviour of these formulations *in vivo* is often governed by an interfacial process. In biological environments, particles interact with various interfaces [16]. The surface activity of the LNCs may have an important influence on their interactions with membranes, including such phenomena as shape changes, vesiculation, membrane disruption and solubilisation.

Many compounds, including pharmacologically active molecules, are amphiphilic—they bear a polar head group (either non-ionic or zwitterionic, anionic or cationic) and a hydrophobic portion [17]. In many ways, colloidal particles resemble surfactant molecules [18]. Although nanoparticles with a suitable size and surface chemistry may strongly adsorb at interfaces, there is an ongoing debate whether they can reduce the interfacial tension [18,19]. For instance, Okubo [20] showed that polystyrene particles, which yielded crystalline structures at the interface, were capable of reducing interfacial tension; however, silica particles, which were independent of the structure and polystyrene particles with non-yielding crystalline structures, did not affect the surface tension. Particles have been shown to act as stabilizers in many foam and emulsion systems (Pickering emulsions) and could prove economically attractive as replacements for conventional surfactants [21].

Similar to surface active compounds, LNCs contain both hydrophilic and hydrophobic parts in their structure. Moreover, the LNC components, macrogol 15 hydroxystearate (MHS) and lecithin, are known for their surface active properties. The Langmuir balance studies [10,16,22–24] suggest that LNCs are capable of decreasing surface tension. When deposited at the air/water interface, the LNCs form a monolayer, but there are no detailed studies on their surface active properties, particularly on their adsorption from the bulk of the liquid.

There are several methods available for the measurement of surface and interfacial tensions. The pendant or sessile drop technique determines the interfacial tension because small drops/bubbles tend to be spherical due to the predominance of surface forces over gravitational forces acting on them. If the drop size is such that the gravitational effects and surface tension are comparable, the interfacial tension can be determined from measurements of the drop/bubble shape [25]. In a Wilhelmy plate method, a thin plate (usually made of platinum or iridium) is dipped in the liquid perpendicular to the interface and the force exerted on the surface is measured. Both methods measure the equilibrium surface tension. Because the nanocarriers are not expected to form the micelles as do conventional surfactants, the term critical micellar concentration (CMC) used for surfactants can be replaced by saturated interfacial concentration (SIC) referring to the nanocarriers.

The aim of this study was to investigate the capability of LNCs to decrease the surface tension using two techniques: drop tensiometry and the Wilhelmy plate method and to compare the surface active properties of the LNCs with those of macrogol 15 hydroxystearate—the surfactant used for their fabrication. Another important goal of this work was to examine the contribution of other LNC components, including oil (triglycerides) and lecithin, to the surface tension of the LNC dispersion. The final objective was to examine the influence of the LNC composition on surface active properties.

## Materials and methods

### Materials

Lipoid S75-3 (hydrogenated lecithin from soybean) was kindly provided by Lipoid Gmbh (Germany). Kolliphor HS 15 (Solutol HS 15, macrogol 15 hydroxystearate, polyoxyl 15 hydroxystearate; CAS Number: 70142-34-6; molecular weight: 963.24 g/mol) was kindly provided by BASF (Germany). Labrafac CC (caprylic/capric acid triglycerides) was kindly provided by Gattefossé S.A. (France). Sodium chloride, chloroform and all other chemicals and solvents were purchased from Sigma Aldrich (France).

### Preparation of the LNCs

LNCs were prepared following the procedure described by Heurtault et al. [9] at a concentration of 120 mg/ml, and the composition of each formulation is shown in Table 1. The LNC components (macrogol 15 hydroxystearate, lecithin and triglycerides) and NaCl (0.089 g) were weighed, mixed with 3 ml of water and heated to 95–100°C. The sample was cooled to 60°C. Three heating-cooling cycles were employed and during the last cooling cycle at 80–90°C (the temperature at which phase inversion occurred) the system was diluted with cold (~4°C) water to the final volume of 17.5 mL. The dispersions for the surface tension measurements were prepared with an aqueous solution of sodium chloride to ensure constant ionic strength.

**Table 1:** Composition and properties of the LNCs. MMR: mass mixing ratio, PS: particle size (mean ± S.D., n = 3), PDI: polydispersity index (mean ± S.D., n = 3), ZP: zeta potential (mean ± S.D., n = 3).

Formulation codes	Macrogol 15 hydroxystearate/triglycerides MMR	Macrogol 15 hydroxystearate	Lecithin	Caprylic/capric acid triglycerides	PS (nm)	PDI	ZP (mV)
LNC30	2.29	67.75%	2.63%	29.62%	26.7±0.1	0.036±0.004	-7.14±0.33
LNC60	0.82	43.38%	3.85%	52.77%	56.0±1.0***	0.028±0.016*	-6.33±1.32**
LNC100	0.40	27.33%	4.24%	68.42%	100.0±2.2	0.047±0.013	-10.62±1.90
LNC81	0.82	45.12%	0.00%	54.88%	81.0±3.4	0.086±0.015	-1.8±0.40
LNC44	0.82	41.71%	7.43%	50.86%	44.0±0.1***	0.041±0.010*	-12.3±3.0**
LNC40	0.82	40.29%	10.71%	49.00%	40.0±0.3***	0.033±0.010**	-23.4±2.4***

*p<0.05

**p<0.01

***p<0.001 versus LNCs without lecithin (LNC81).

The number of nanocapsules was calculated [13] using the following equation:

$$x=\frac{3\;m}{\rho \pi 4r^{3}}$$

where m is the mass of the triglycerides, ρ is the density of the triglycerides (945 g/L), r is the radius of the nanocapsule, and x is the number of the nanocapsules.

### Characterization of the LNCs

The intensity-averaged particle diameter and the polydispersity index of the LNCs were determined by dynamic light scattering (DLS) with 173° backscatter detection. The electrophoretic mobility values measured by laser Doppler velocimetry (LDV) were converted to a zeta potential by the Smoluchowski equation. Both DLS and LDV measurements were done on a Zetasizer nano-series Nano-ZS fitted with a 633 nm laser (Malvern Instruments, UK). The measurements were performed at an LNC concentration of 3 mg/mL after dilution with MilliQ water. Each analysis was conducted at 25°C in triplicate.

### Preparation of the macrogol 15 hydroxystearate solution and its mixture with other LNC ingredients

The following solution and mixtures were prepared: (1) a macrogol 15 hydroxystearate solution, (2) a mixture of macrogol 15 hydroxystearate solution and hydrogenated lecithin, (3) a mixture of macrogol 15 hydroxystearate and triglycerides and (4) a mixture of macrogol 15 hydroxystearate, hydrogenated lecithin and triglycerides. The quantities of ingredients in the mixtures/solution were the same as in the LNC60 formulation. The lecithin was solubilized in chloroform. The remaining ingredients (macrogol 15 hydroxystearate and triglycerides) and NaCl were weighed. All ingredients were dispersed in water and left under magnetic stirring overnight for the chloroform to evaporate.

### Surface tension measurement by the Wilhelmy plate method

A platinum plate was attached to a laboratory microbalance so that it was perpendicularly oriented to the interface. The liquid level was raised until it just touched the hanging plate. The mass recorded on the balance was noted, and the surface tension (γ) was calculated using the Wilhelmy equation:

$$\gamma =\frac{m∙g}{l∙cos\theta }$$

where m is the mass measured by the balance, g is the standard acceleration due to gravity (g = 9.81 m/s^2^), l is the wetted perimeter of the Wilhelmy plate and Ɵ is the contact angle between the liquid phase and the plate.

The wetted perimeter (l) was calculated using the following equation:

l=2w+2d

where w is the plate width (w = 2 cm) and d is the plate thickness (it was assumed that d = 0 cm because of the negligible thickness of the plate). Platinum was chosen as the plate material because it is chemically inert and easy to clean. Additionally, platinum can be optimally wetted because of its high surface free energy and generally forms a contact angle (Ɵ) of 0° (cosƟ = 1) with liquids.

### Surface tension measurement by drop tensiometry

The surface tension was also measured using a drop tensiometer (Tracker, ITConcept, Longessaigne, France). A stainless steel, U-shaped needle with a gauge (G18) and flat-cut tip connected to a 0.25 mL Exmire microsyringe containing air was dipped into the sample and placed in a 1x2x4.3 cm cuvette made of optical glass (Hellma, France) so that the tip was covered by the aqueous phase. The contents of the cuvette were illuminated by a 10-W halogen lamp. A rising air bubble with a controlled volume of 5 μL (approximately 12.5 mm^2^) was delivered by pushing the piston of the syringe by a DC motor with a 100 count per revolution optical encoder (Maxon motor, Switzerland). After the formation of the axisymetric bubble, its profile was digitised and analysed by a CDD camera that was coupled to a video image profile digitiser board (Imaging Technology, model PCVision Plus) and connected to a personal computer. The bubble profile was processed by a WINDROP software package using the Laplace equation:

$$\frac{1}{x}\frac{d}{dx}(x\;sin\theta )=\frac{2}{b}-Cz$$

where x and z are the Cartesian co-ordinates at any point of the bubble profile, b is the curvature radius at the bubble apex, Ɵ is the angle of the tangent to the bubble profile and C is the capillarity constant.

The capillarity constant can be calculated using the following equation:

$$C=\frac{g∙∆\rho }{\gamma }$$

where ∆ρ is the difference between the densities of the aqueous phase and the air, γ is the surface tension and g is the standard acceleration due to gravity (g = 9.81 m/s^2^) [26].

The surface tension and surface area were simultaneously calculated and recorded in real time.

### Calculation of surface excess concentration, % of surface coverage, surface pressure, area per molecule and critical micelle concentration/saturated interfacial concentration

The surface excess concentration (Г_2_) was calculated using the Gibbs adsorption equation applicable to dilute solutions:

$${Г}_{2}=-\frac{c}{RT}\frac{d\gamma }{dc}$$

where γ is the surface tension, c is the concentration of macrogol 15 hydroxystearate, T is the absolute temperature (in kelvins) and R is the gas constant (R = 8.314 J mol^-1^ K^-1^).

The surface coverage percentage was calculated by dividing the surface excess concentration at a given concentration of macrogol 15 hydroxystearate in the bulk by a maximal surface excess concentration multiplied by 100%.

The surface pressure (π) is the difference between the surface tension of the clean surface (γ_0_) and that of the surfactant-covered surface (γ_m_):

$$\pi =\gamma_{0}-\gamma_{m}$$

The area (A) occupied by each macrogol 15 hydroxystearate molecule was calculated using the following equation:

$$A=\frac{1}{N_{A}{Г}_{2}}$$

where Г_2_ is the value of surface excess concentration calculated from the Gibbs equation and N_A_ is the Avogadro constant (N_A_ = 6.022x10^23^ mol^-1^).

The critical micelle concentration and saturated interfacial concentration were determined from the plots of surface tension versus logarithm of surfactant concentration. The CMC/SIC was taken as the concentration at the point of intersection of the two linear portions of the γ versus log c plots. The slope of the linear portions of each curve in the plot was determined by the method of least mean squares. In some cases, it was not possible to determine the CMC/SIC based on those calculations because the plots of surface tension versus concentration showed irregularities in shape. In these cases, the point of the curve at which a sharp change of slope occurred was taken as the CMC/SIC.

### Statistical analysis

The statistical significance in the differences between samples was determined using a one-way analysis of variance (ANOVA). The differences were considered to be significant at *p* < 0.05. Statistical data treatment was used to determine the influence of lecithin on the properties of the LNCs.

## Results

### Influence of the composition on LNC properties

Varying the quantities of the LNCs ingredients, particularly MHS and triglycerides, yielded particles with sizes between 27 and 100 nm (Table 1). The higher the surfactant/oil mass mixing ratio, the smaller the diameter of the LNCs. Those formulations were characterized by a homogenous size distribution (PDI below 0.1) and slightly negative zeta potential.

Nanocapsules composed solely of oil and MHS have been successfully obtained, similar to those previously reported [2]. The incorporation of lecithin, a lipophilic surfactant, considerably decreased the size of the LNCs from 81 to 56 nm. A further increase in the lecithin concentration from 3.85 to 7.43% or 10.71% resulted in a further decrease in particle size from 56 to 44 nm and 40 nm, respectively. The incorporation of lecithin also considerably increased the zeta potential (in modulus). A linear correlation was found between the lecithin concentration in the LNCs and LNC properties, such as particle size and zeta potential (R^2^ = 0.912 and R^2^ = 0.9408 for the particle size and zeta potential, respectively).

### Surface activity of the LNCs and macrogol 15 hydroxystearate measured by drop tensiometry

**Fig 1:**
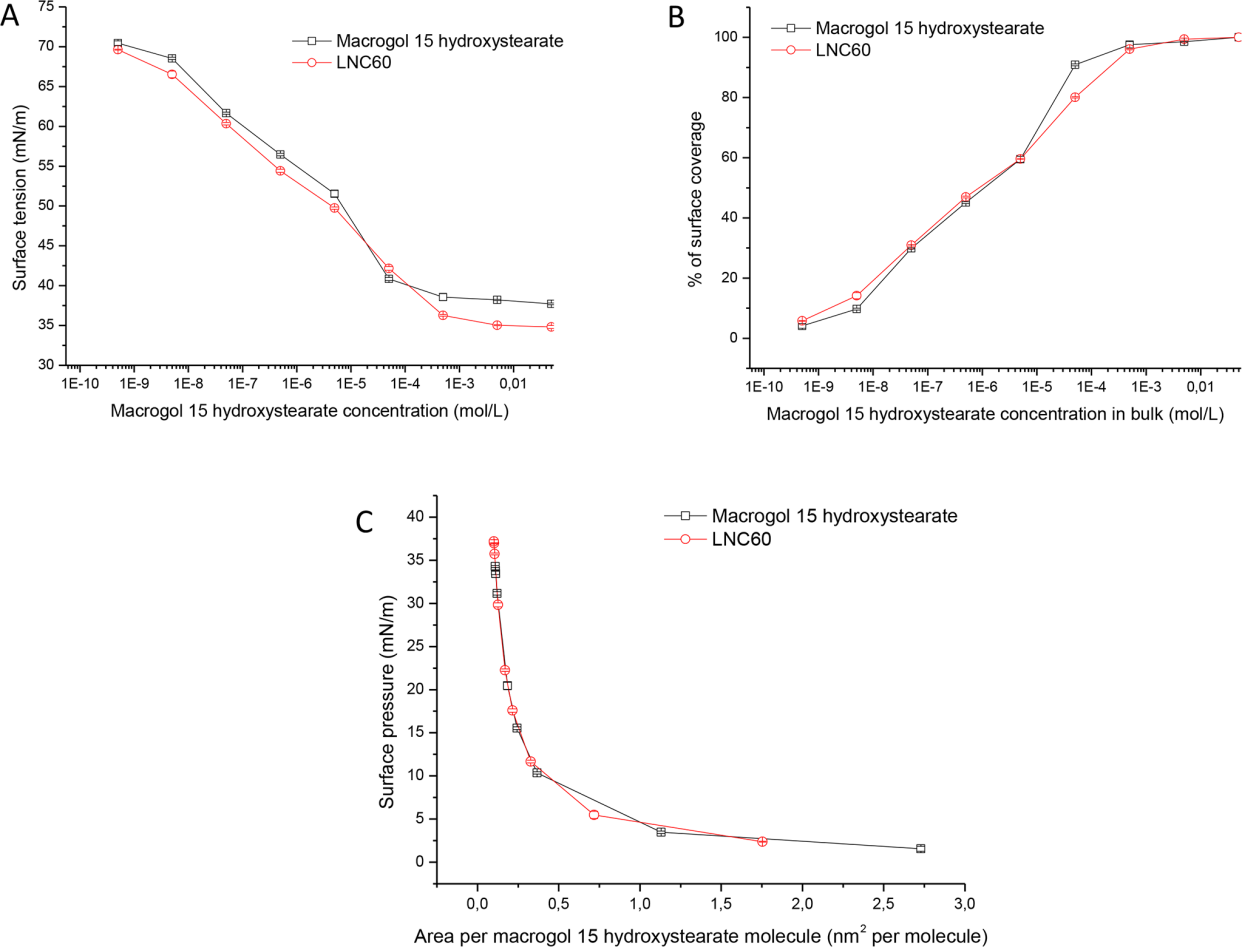
Surface tension as a function of the MHS concentration (A) surface coverage percentage as a function of MHS concentration, (B) surface pressure versus area per MHS molecule and (C) the MHS solution and LNC dispersion as determined by drop tensiometry.

Fig 1A shows the surface tension isotherms for MHS and LNC60 measured using a drop tensiometer. The isotherm may be divided into two regions. In the first region, there is a decrease in surface tension with increasing surfactant or LNC concentration. At low MHS or LNC60 concentration only slight changes in surface tension were detected, whereas at higher concentrations the further addition of surfactant/LNCs drastically decreased the surface tension. In the second region of surface tension isotherms, the line became horizontal because further additions of surfactant or LNC60 were not accompanied by a decrease in surface tension. This is because the surface became saturated.

Because the particles do not assemble in the same way as surfactant molecules, which aggregate and form micelles [18], we used the saturated interfacial concentration (SIC) for the LNC formulations instead of the critical micellar concentration (CMC). The sharp change in slope in the surface tension isotherms corresponds to CMC/SIC. The obtained CMC value of 0.073±0.0056 mg/mL (0.076±0.006 mM) for MHS agrees well with the manufacturers data (CMC between 0.005 and 0.02%, equivalent to 0.05–0.2 mg/mL) (Table 2). A 5-10-fold increase in the SIC of MHS has been observed when this surfactant was incorporated into the LNCs compared with the CMC of free MHS.

**Table 2:** Critical micellar concentration (CMC), saturated interfacial concentration (SIC) and the limiting surface tension of the MHS solution and LNC dispersion as determined by drop tensiometry. The CMC/SIC is the concentration at which a sharp change in the slope has occurred. The concentrations (given in brackets) correspond to the calculated CMC/SIC based on the slope of the surface tension isotherms. The limiting surface tension is the lowest surface tension observed for a given system regardless of the bulk concentration.

Sample	CMC/SIC in mM of macrogol 15 hydroxystearate	CMC/SIC in mg of LNCs/mL	Limiting surface tension (mN/m)
Macrogol 15 hydroxystearate	0.050 (0.076±0.006)	N/A	37.72±0.10
LNC60	0.500 (0.388±0.073)	0.8605±0.1618	34.81±0.19

Interestingly, the LNCs were capable of decreasing the surface tension to a lower value than MHS (37.72±0.10 mN/m and 34.81±0.19 mN/m for MHS and LNC60, respectively). This result suggests that both MHS and the other components of the LNCs contribute to the decrease in surface tension of the LNC dispersion. Fig 1B shows the surface coverage as a function of MHS concentration in the bulk liquid. It was observed that the surface coverage curves were similar in shape for both the MHS solution and LNC60 dispersion, but in the case of the surfactant solution, the surface coverage was achieved for lower MHS concentrations in the bulk. At 0.049 mM MHS, a surface coverage of 90% was achieved, whereas at the same MHS concentration in the LNC60 dispersion (equivalent to 0.111 mg/mL LNC60), a surface coverage of 80% was achieved. Fig 1C shows the surface pressure-area per MHS molecule isotherm for the MHS solution and LNC60 dispersion. The shape of the isotherms and the area per molecule at the surface saturation concentration were similar for both systems (0.10–0.11 nm^2^ per molecule), although the LNC dispersion molecules of other ingredients (triglycerides, lecithin) were also present in the system.

**Fig 2:**
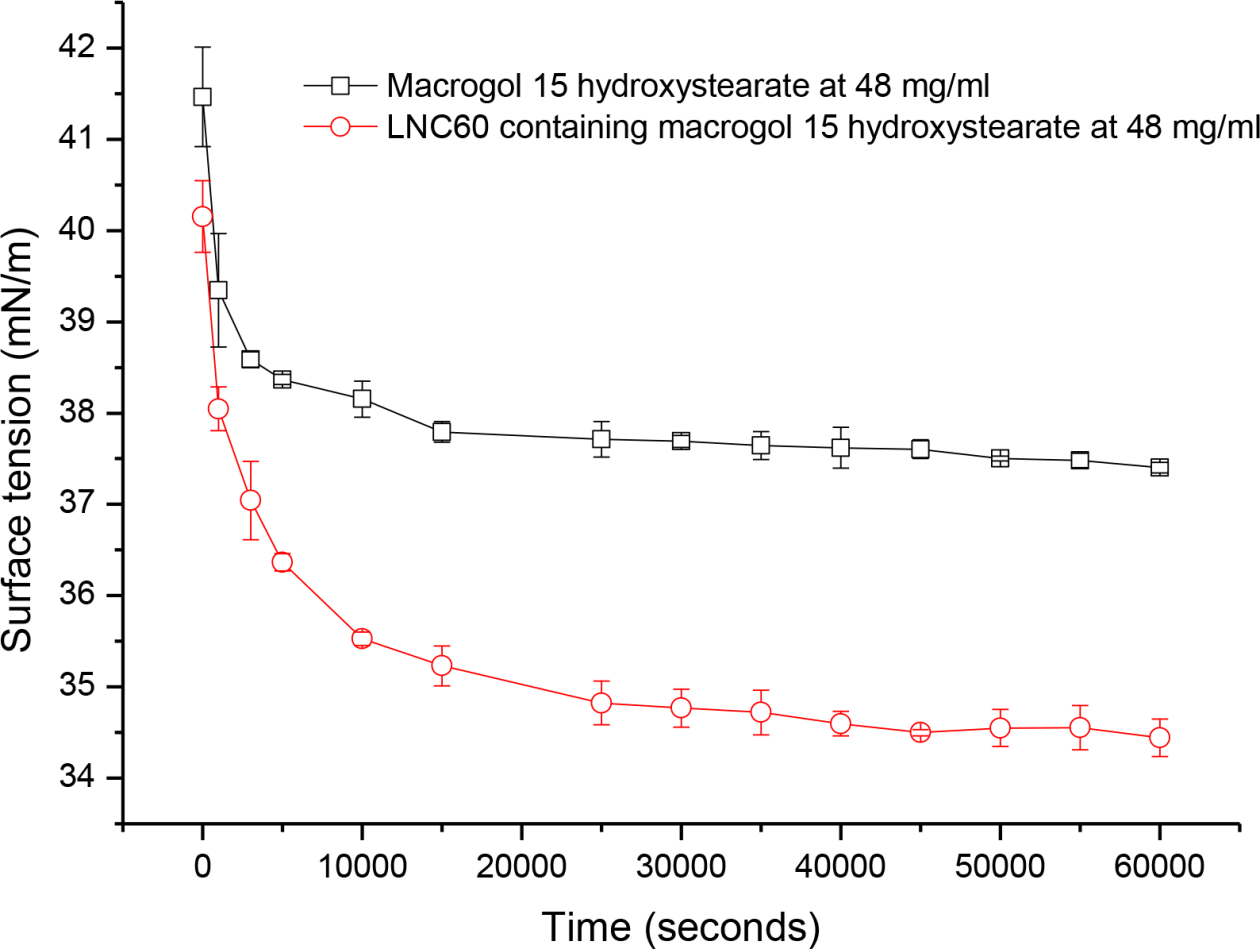
Surface tension versus time of the MHS solution (50 mM) and LNC dispersion containing 50 mM MHS as determined by drop tensiometry.

Fig 2 shows changes in surface tension as a function of time for both MHS and LNC60 dispersions. A sharp reduction in the surface tension was observed in the first minutes of the experiment after the formation of air bubbles in both the MHS solution and LNC dispersion, followed by a gradual decrease. After reaching the equilibrium surface tension, no change in surface tension was observed. The time necessary to achieve an equilibrium in surface tension was much longer in the LNCs (approximately 7 hours) than in the MHS solution (approximately 4 hours). This time was further increased with a decrease in MHS or LNCs concentration (5–6 hours and 9 hours for MHS and LNC60, respectively (not shown)).

### Surface activity of the LNCs and macrogol 15 hydroxystearate measured by the Wilhelmy plate method

**Fig 3:**
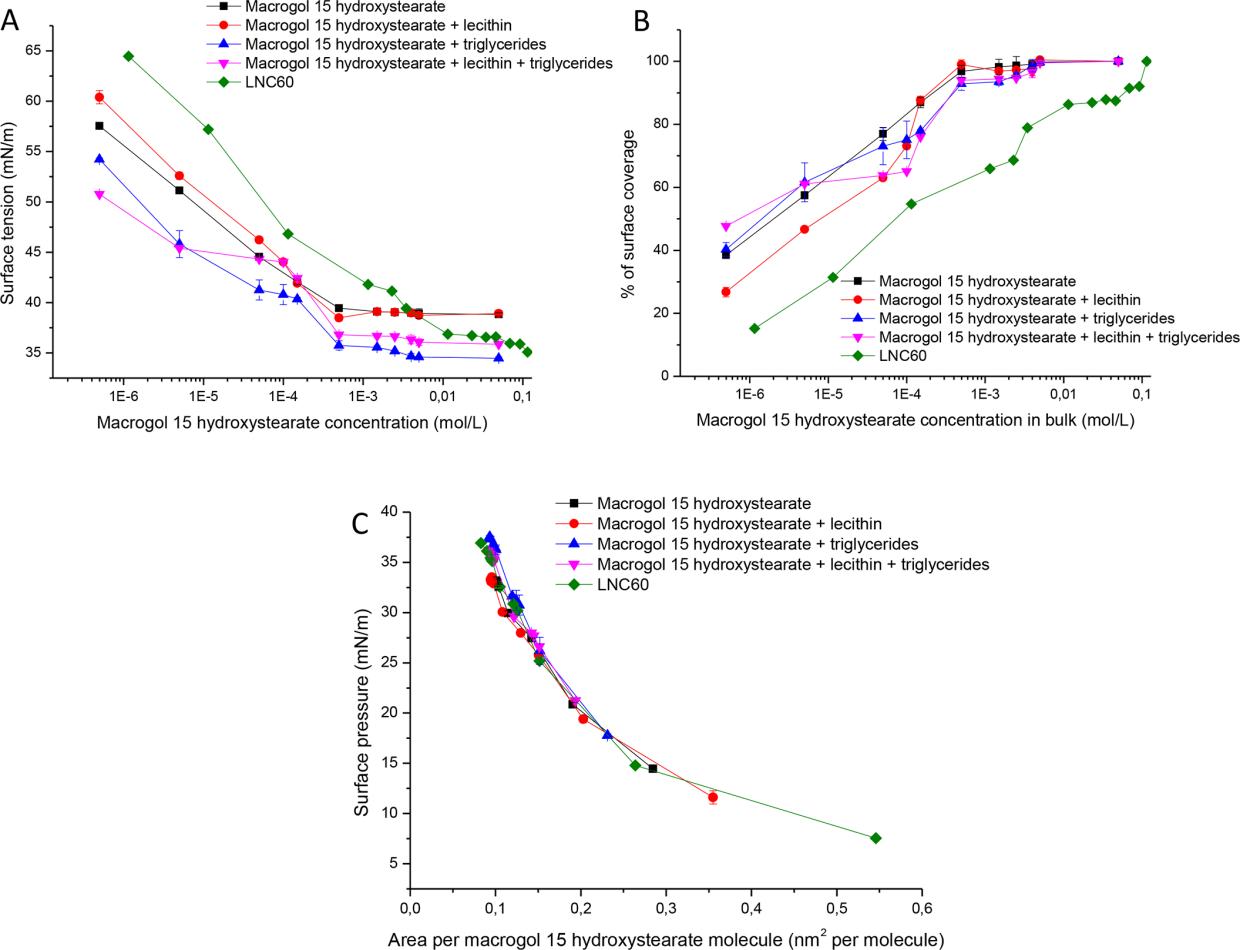
Surface tension as a function of the MHS concentration (A)surface coverage percentage as a function of MHS concentration, (B) surface pressure versus area per MHS molecule and (C) the MHS solution, LNC dispersion and binary and ternary mixtures of LNC components as determined by the Wilhelmy plate method.

Fig 3A shows surface tension versus concentration plots for MHS, LNC60 and binary and ternary physical mixtures of the LNC components, and Table 3 shows CMC/SIC values. The MHS isotherm obtained by the Wilhelmy plate method has a similar shape to that obtained by drop tensiometry in the sense that it exhibits a sharp change in slope at the CMC, but the CMC value was 5-10-fold higher than that measured by drop tensiometry. The measurement by the Wilhelmy plated method lasted approximately 15 minutes—it was impossible to perform long-time measurements due to liquid evaporation effects. The lecithin/MHS mixture indicated a decrease in the surface tension to values similar to the unmixed MHS values (39.10±0.13 mN/m and 38.83±0.17 mN/m, respectively), but the isotherm of the binary mixture exhibited a minimum surface tension of 38.41±0.31 mN/m near the SIC value. In contrast to the lecithin/MHS mixture, the binary mixture composed of MHS and triglycerides exhibited the lowest surface tension (34.5±0.14 mN/m) from all tested systems. In addition, concerning the SIC (0.500 mM of MHS), another change in the slope was observed at a lower concentration (0.050 mM of MHS), with the isotherm forming a second horizontal line at concentrations ranging from approximately 0.050–0.149 mM MHS. Similarly, the MHS/triglycerides binary mixture (a ternary mixture) also produced an important decrease in surface tension compared with that of MHS (35.86±0.11 mN/m and 38.83±0.17 mN/m, respectively) and a second horizontal line in surface tension isotherm at the concentrations from 0.005–0.100 mM MHS.

**Table 3:** Critical micellar concentration (CMC), saturated interfacial concentration (SIC) and the limiting surface tension of the MHS solution, LNC dispersion and binary and ternary mixtures of LNC components as determined by the Wilhelmy plate method. The CMC/SIC is the concentration at which a sharp change in the slope has occurred. The concentrations (given in brackets) correspond to the calculated CMC/SIC based on the slope of the surface tension isotherms. The limiting surface tension is the lowest surface tension observed for a given system regardless of the bulk concentration.

Sample	CMC/SIC in mM of macrogol 15 hydroxystearate	Limiting surface tension (mN/m)
Macrogol 15 hydroxystearate	0.500 (0.339±0.016)	38.83±0.17
Macrogol 15 hydroxystearate + lecithin	0.500 (0.329±0.042)	39.10±0.13
Macrogol 15 hydroxystearate + triglycerides	0.500	34.50±0.14
Macrogol 15 hydroxystearate + lecithin + triglycerides	0.500	35.86±0.11
LNC60	5.000	35.01±0.08

The LNC60 formulation was capable of reducing the surface tension to a significantly lower value (35.01±0.08 mN/m) than MHS or its binary mixture with lecithin. This is in good agreement with results obtained with the drop tensiometry method.

Fig 3B shows that surface saturation occurs at much lower MHS concentrations in bulk MHS and its binary and ternary mixtures compared with that observed for LNC60. The surface coverage was 98% at 0.149 mM MHS and 0.500 mM for its binary mixture with lecithin. The surface coverage percentages of 92–93% at 0.500 mM were attained for both the binary (MHS/triglycerides) and ternary mixtures, whereas a surface coverage of 92% was achieved at 69.0 mM MHS for LNC60.

The surface pressure-area isotherms for the LNCs, macrogol 15 hydroxystearate and binary and ternary mixtures had similar shapes (Fig 3C). The area per molecule is similar for all systems and was also similar to the results obtained by drop tensiometry. However, the shape of the isotherms obtained by the Wilhelmy plate method was different than those obtained by drop tensiometry. The former produced expanded liquid monolayers, whereas the latter produced compressed liquid monolayers.

### Influence of the composition on LNC surface activity

**Fig 4:**
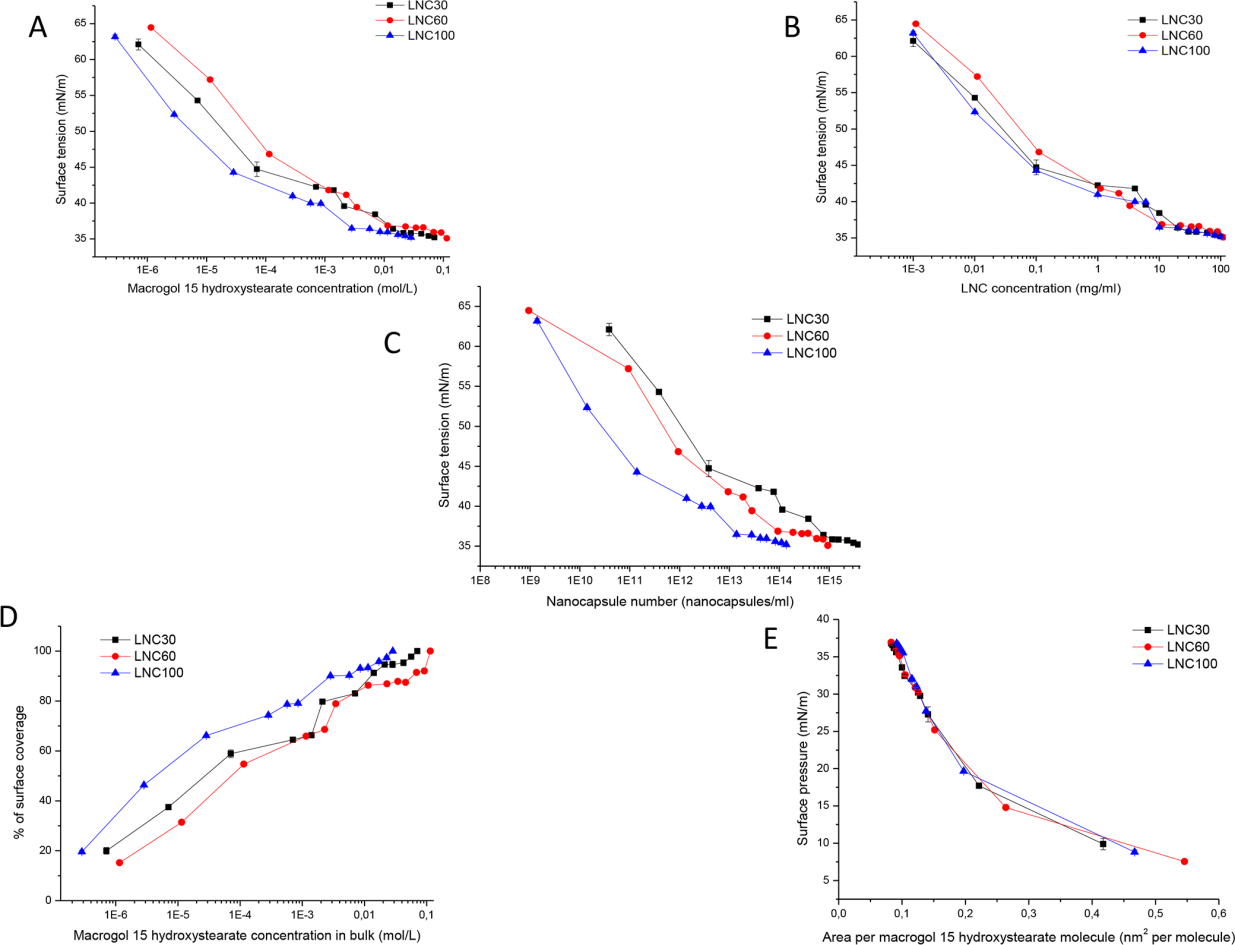
Surface tension as a function of MHS concentration (A) LNC concentration, (B) nanocapsule number, (C) surface coverage percentage as a function of MHS concentration, (D) surface pressure versus area per MHS molecule and (E) LNC formulations containing different mixing ratios of MHS/triglycerides as determined by the Wilhelmy plate method.

Fig 4 shows surface tension isotherms as a function of MHS concentration (Fig 4A), LNC concentration (Fig 4B) and nanocapsule number (Fig 4C) of LNCs with an MHS/triglycerides mixing ratio and consequently a different particle size. All formulations were capable of decreasing the surface tension to similar values (35.19±0.18 mN/m, 35.01±0.08 mN/m and 35.18±0.05 mN/m for LNC30, LNC60 and LNC100, respectively) (Table 4). The SIC can be ranked in the following order (LNC100>LNC60>LNC30) when the MHS concentration is considered. They may be similarly ranked if the particle number is considered (Fig 4C). However, when the LNC concentration is considered, all formulations showed comparable SIC. The surface saturation was reached at a lower concentration of MHS for LNC100, then for LNC30 and the highest for LNC60 (Fig 4D). More than 90% of the surface coverage was achieved at 2.85 mM MHS for LNC100, 14.15 mM MHS for LNC30 and 69.0 mM MHS for LNC60. The surface pressure-area per molecule isotherm had a similar shape and similar area per MHS molecule for all three LNC formulations (Fig 4E).

**Table 4:** Saturated interfacial concentration (SIC) and limiting surface tension of the LNC formulations determined by the Wilhelmy plate method. The SIC is the concentration at which a sharp change in the slope has occurred. The limiting surface tension is the lowest surface tension observed for a given system regardless of the bulk concentration.

Sample	SIC in mg of macrogol 15 hydroxystearate/mL	SIC in mg of LNCs/mL	Limiting surface tension (mN/m)
LNC30	21.185	30.13	35.19±0.18
LNC60	5.000	11.08	35.01±0.08
LNC100	2.849	10.04	35.18±0.05
LNC81	5.000	10.67	36.78±0.03
LNC44	5.000	11.55	36.46±0.04
LNC40	5.000	11.95	36.26±0.08

**Fig 5:**
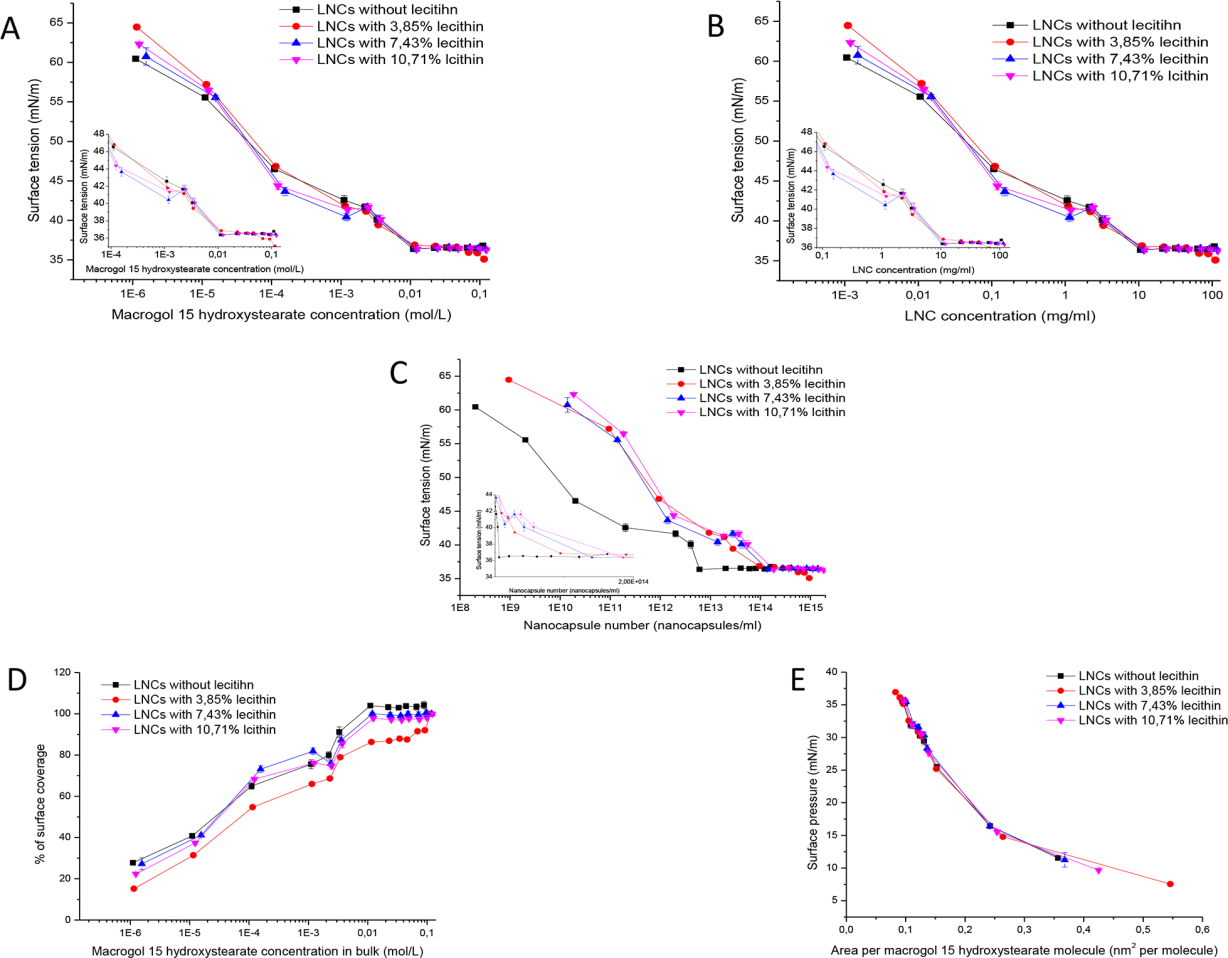
Surface tension as a function of MHS concentration (A) LNC concentration, (B) nanocapsule number, (C) surface coverage percentage as a function of MHS concentration, (D) surface pressure versus area per MHS molecule and (E) LNC formulations containing different quantities of lecithin as determined by the Wilhelmy plate method.

Fig 5 shows surface tension isotherms as a function of MHS concentration (Fig 5A), LNC concentration (Fig 5B) or nanocapsule number (Fig 5C) of LNCs containing different quantities of lecithin. The removal of lecithin or an increase in lecithin concentration to 7.4 or 10.7% yielded LNCs capable of decreasing the surface tension to 36.3–36.8 mN/m. These values are significantly larger than those observed for LNC60 containing 3.85% lecithin. The SIC was not affected either by the presence/absence or by the quantity of lecithin at MHS/TG MMR = 0.82 (Table 4). Interestingly, in the LNC isotherms containing a high lecithin percentage (7.4 or 10.7%), a minimum was observed at 1.19 mM MHS. This minimum was also observed in the surface coverage isotherms (Fig 5D). A minimum in the surface tension isotherm was also observed for the binary mixture of lecithin and MHS but it occurred at SIC. When the LNC number is considered, the shape of the lecithin-LNCs isotherms was similar, whereas the LNCs without lecithin show a larger surface activity particularly at lower particle numbers. Because the number of particles was estimated theoretically from the size and mass of triglycerides, these results should be interpreted with caution. LNC40, LNC44 and LNC90 provided surface saturation at lower concentrations than LNC60 (Fig 5D). The shape of the surface pressure-area per molecule isotherm and surface area were similar in all LNC formulations (Fig 5E).

## Discussion

### Influence of the composition on LNC properties

MHS, the main surfactant used in the fabrication of the LNCs, consists of polyglycol mono- and di-esters of 12-hydroxystearic acid and approximately 30% free polyethylene glycol. It was used as a non-ionic solubiliser and an o/w emulsifying agent (the HLB value was between 14 and 16). The MHS surfactant molecules are soluble in water and forms a clear solution (manufacturer’s data). The solubility of MHS in water decreases with increasing temperature, which is important for the fabrication of the LNCs because it enables the phase inversion from an o/w to w/o emulsion upon heating. The cloud point (the temperature at which MHS is no longer soluble in water) is 63°C [3]. MHS and other polyoxyethylene-type non-ionic surfactants become more lipophilic at higher temperatures due to the dehydration of polyoxyethylene chains resulting from the breakdown of hydrogen bonds with water molecules [8]. Below the cloud point non-ionic emulsifiers in water form micelles, which are composed of approximately 100 molecules. The dispersion remains transparent because these micelles are much smaller than the wavelength of light. Although the micellar solution is transparent, the formation of micelles is a phenomenon similar to a phase separation [27,28].

LNCs with the sizes ranging from 25 to 100 nm have been described to date. It has been shown that the properties of the LNCs strongly depend on the proportions of the ingredients [3,8,9,13]. LNCs contain a small quantity of lecithin (usually less than 10%), which is an important factor in determining if the LNCs can be obtained. In addition, the size of the prepared LNCs is dependent on the quantity of oil (triglycerides) and MHS. The presence of the PEGylated surfactant (e.g., MHS) is necessary for phase inversion. Although the nanocapsules composed solely of MHS and lecithin can be obtained, it has been shown that they are not stable at the air/water interface [10]. Co-surfactants, such as lecithin have been shown to stabilize the LNCs [3,10]. The incorporation of lecithin has decreased the size of the LNCs and increased the zeta potential in modulus [2,29]. Lecithin is an ampholytic surfactant that contains phosphate and amino groups and has an isoelectric point of 4.15 [30]. The negative values of the zeta potential indicate that the charge of the phosphate groups predominated under the examined conditions (pH = 6), similar to previous reports [2]. Despite low values of zeta potential LNCs showed good stability, which may be attributed to steric stabilization due to the presence of PEG moieties (MHS) on the surface of the nanocarriers.

### Surface activity of LNCs and their ingredients

The surface activity of the LNCs and their ingredients was examined using two methods: drop tensiometry and the Wilhelmy plate method. The turbidity of the nanoparticulate dispersion depends on the particle size and particle number/concentration [31,32]. The larger LNCs (80–100 nm) formed turbid dispersions at higher concentrations, as did the mixtures of the LNC ingredients. For this reason and due to the long duration of drop tensiometry measurements, most samples were only tested by the Wilhelmy plate method, and drop tensiometry was solely used to compare the properties of the LNC60 and MHS.

The CMC/SIC of LNC60 was 5–10 times higher than that of MHS, as determined by both drop tensiometry and the Wilhelmy plate method. The CMCs/SICs of the mixtures of LNC ingredients were the same as those of MHS. Apparently, the free MHS molecules were mainly responsible for the decrease in surface tension. The LNC dispersion may have contained free MHS molecules, which were not attached to the LNC surface. These molecules may be responsible for the decrease in surface tension of the LNC dispersions. The remaining MHS molecules were attached to the surface of the LNCs, which prevented their adsorption at the interface, and consequently numerous MHS molecules in the system were required for interface saturation. It is known that NP surface adsorption decreases the surfactant efficiency by lowering the interfacial tension and concurrently prevents nanoparticle aggregation [19]. Because the LNC surface is larger than that of the emulsions formed by the ingredient mixtures, it is expected that the number of MHS molecules entrapped on the LNC surface will be higher. Interestingly, the CMCs/SICs obtained by drop tensiometry were 10 times smaller than those obtained by the Wilhelmy plate method. As discussed later in more detail, the differences may be explained by different measurement durations.

The molecular film at the air/water interface acts like a two-dimensional analogue to normal matter in that it may exist in different physical states, which in certain ways resemble solids, liquids and gases [33]. Both drop tensiometry and Wilhelmy plate measurements have revealed that the shape of the surface pressure isotherms versus area per MHS molecule of MHS and LNC60 was the same for a given method. Moreover, the Wilhelmy plate measurements showed that binary and ternary mixtures and all tested LNC formulations produced isotherms with the same shape as those of MHS and LNC60. When adsorbed from the bulk, the MHS molecules were mainly present on the surface and formed a continuous phase. This is in contrast to the results obtained by Heurtault et al. [16], which showed different isotherm profiles for the LNCs, MHS and the mixture of the LNC ingredients. Unlike in our case, a Langmuir balance was utilized, and the samples were deposited on the surface and not adsorbed from bulk. The shape of the LNC isotherm observed by Heurtault et al. [16] was similar to the MHS isotherm but was expanded. The triglycerides (not tested in our study) at a large area per molecule showed typical behaviour to that of the liquid, but after compression they exhibited a phase transition to a more condensed monolayer. The lecithin produced a typical condensed film—the film pressure remained very low at high film areas and abruptly increased when the molecules became tightly packed during compression. In a film of a ternary mixture of the LNC ingredients, the presence of both triglycerides and lecithin was evident. Therefore, it was concluded that the composition of the monolayer depends on the deposition. The adsorption or desorption of the surface active material on or out of the interface followed three steps: (i) diffusion and/or convection between a deeper layer of the solution/dispersion and sub-surface layer immediately adjacent to the interface, (ii) adsorption and/or desorption between the sublayer and interface and (iii) rearrangement of adsorbed molecules at the interface. The diffusion was driven by a concentration gradient, the adsorption/desorption was driven by the chemical potential and the rearrangement may be caused by reorientation, complex formation, phase transition or formation of a two-dimensional micelle structure [34]. In the case of the surface deposition, the first stage did not occur, which may explain the differences observed between the deposition in the bulk or on the surface.

The mixtures containing triglycerides were capable of reducing the surface tension to a larger extent than MHS. Similarly, the LNCs showed a lower limiting surface tension than MHS as measured by both drop tensiometry and the Wilhelmy plate method. This finding may suggest that the triglyceride molecules, although they do not form a continuous phase on the surface, are present in the monolayer formed by MHS and significantly contribute to the reduction in surface tension. Another explanation is that caprylic/capric acid triglycerides contain some impurities, e.g., mono- and diglyceride molecules or free fatty acids, which are known for their surface activity and may be present in the monolayer.

The binary mixture of MHS and lecithin had a similar limiting surface tension as the MHS, and their isotherms had very similar shapes with the exception that the isotherm of the binary mixture exhibited a minimum surface tension around the SIC. The isotherms with a similar shape and minimum surface tension were observed for the sodium dodecylsulfate solution likely because of impurities present in the surfactant solution [35]. It was concluded that at high concentrations (above the SIC), the lecithin molecules are excluded from the interface, despite the surface active properties of the phospholipids.

A question arises as to what happens to the nanocapsules when they reach the surface. The density and structure of the nanoparticle film at the gas/liquid interface depends on the equilibrium between the attractive and repulsive forces between the nanoparticles and the exchange of nanoparticles between the interface and bulk of the liquid. Whilst electrostatic forces serve as the repulsive forces for the charged nanoparticles, van der Waals forces may serve as the attractive forces, particularly for closely spaced nanoparticles [36]. In general, the forces responsible for the interactions between particles in the bulk are also operative in particulate monolayers on the surface [18]. Whilst the estimated distances between the nanoparticles are based on the bulk concentration and are relatively large (>10–100 nm diameters depending on the concentration), shorter distances may occur if the nanoparticles accumulate and assemble at the liquid-gas interface [36]. Therefore, nonelectrostatic forces, such as hydrophobic, polar and hydrogen bonding interactions, become more important in the two-dimensional ordering at the interface compared with the bulk [20]. Such interactions may lead to a reduction in the repulsion and consequently particle aggregation and destruction. For instance, it has been demonstrated that phospholipid vesicles can disrupt on contact with an air/water interface. This disruption may allow the flow of phospholipid monomers and their deposition at the interface thus forming a monolayer and consequently decreasing the surface tension [37–39]. Similarly, emulsions or high density lipoproteins (HDL) have been shown to destabilize at the interface thereby releasing their components, such as triglycerides [37,40]. It has been suggested that during the spreading of the LNCs on the air/water surface, a small amount of MHS located at the nanocapsule surface was released from the initial structure, but this did not lead to the destruction of the nanocapsules [16]. It has been suggested that in lipid nanocapsules a sufficiently rigid shell prevents their opening [16]. Indeed, it has been shown that LNC deformation is possible to some extent without the destruction of the particle structure [13]. Luo and Dai [41] have conducted simulations and found that the surfactants and nanoparticles compete for adsorption at liquid-liquid interfaces and that as the surfactant concentration increased, the nanoparticles desorbed from the interface. The MSH molecules reach the interface faster than the LNCs, and when adsorbed at the surface, the MHS molecules may repel the approaching LNCs. This effect may contribute to the similarities between the LNC and MHS films on aqueous substrates.

As mentioned, the isotherm shapes produced by the Wilhelmy method were different that those obtained by drop tensiometry. The differences between these two methods may be explained by different measurement durations. Drop tensiometry enables a measurement of equilibrium surface tension, which corresponds to the equilibrium or maximum biosurfactant concentration at the drop-air interface. The minimum surface tension is observed at very long times in measurements of surface tension versus time [42]. In contrast, a measurement using the Wilhelmy plate method is much shorter (approximately 15 minutes). As shown in Fig 2, more than 15 minutes is required for the MHS molecules or the LNCs to reach the interface, particularly at lower concentrations. Moreover, this study confirms the well-known fact that particle adsorption at interfaces is slow compared with surfactant molecules [18].

Furthermore, the extrapolated limiting surface area per MHS molecule (approximately 0.1 nm^2^) was the same for all samples tested by both methods. This low value may suggest that at high concentration the MHS molecules stand upright and are closely packed together. However, as indicated by the formation of a liquid film, the MHS molecules may be lying along the surface at lower concentrations. The area per molecule for SDS was 0.47 nm^2^ [43], and a limiting surface area of approximately 0.20 nm^2^ was found for fatty acids [33]. However, the limiting area per MHS molecule may be higher than that obtained from the calculations. Firstly, MHS contains approximately 30% free polyethylene glycol molecules, which are hydrophilic and may not be located in the surface. Second, it is possible that even below the CMC, not all MHS molecules are present on the surface. A portion of them may remain in the bulk.

### Influence of composition on the LNC surface activity

Certain of the physical and biological properties of the nanocarrier can be markedly changed by adding a new ingredient or changing the properties of the ingredients. For instance, incorporation of fatty acids or monoglycerides yielded LNCs with antibacterial properties [29]. Changes in the quantities of the LNC ingredients (particularly MHS/triglycerides mixing ratio) affects the properties of the LNCs, such as particle size but also their capability to encapsulate drug molecules [3]. The composition of the LNCs affects their redispersibility after freeze-drying [44], their nebulization [3] and their interactions with cells and uptake [12]. Therefore, it is very important to examine if the composition of the LNCs affects their ability to decrease the surface tension. Indeed, the studies by [10] have shown that the content of MHS, triglycerides and lecithin influence the kinetics of film formation after surface deposition.

Increasing the nanoparticle size can significantly increase the desorption energy, which leads to larger surface coverage and therefore larger interfacial tension reductions [45,46]. This may explain why at a lower SIC, the same number of large nanocapsules provides a higher reduction in surface tension compared with smaller LNCs.

The SIC depended on the mass mixing ratio of MHS/triglycerides but not on the presence of lecithin. It has been confirmed that after the deposition of lipid nanocapsules on the surface, lecithin was neither released and nor located at the interface [16]. The lecithin molecules were found to stabilize the LNC structure at the interface [10]. Two populations of the LNCs (with and without phospholipid molecules) were detected. The lecithin prevented the release of molecules from the LNCs. It has been suggested that after interface spreading a rapid dissociation of the unstable LNCs containing smaller quantity of lecithin occurs, which led to the rapid release of triglyceride molecules, and a small quantity of MHS molecules instantaneously formed a liquid-expanded monolayer. After the deposition, the stable population of LNCs with a large quantity of lecithin remained intact and slowly released only the single MHS molecules weakly attached to the shell [10]. The adsorption of the LNCs on the surface is particularly important when the interface area is largely increased compared with that of the bulk. For instance, it has been observed that the size of the LNCs significantly increased after nebulization. This increase in particle size was attributed to adsorption of the LNCs on the surface of aerosol droplets [3].

## Conclusions

The properties of LNCs strongly depend on their composition. The size of LNCs was mainly influenced by the quantity of the MHS in the LNCs. However, the addition of lecithin significantly affected both the particle size and zeta potential.

The LNCs decreased the surface tension values more than the MHS or MHS/lecithin mixtures, as confirmed by both drop tensiometry and the Wilhelmy plate method. The CMC/SIC values measured by the Wilhelmy plate method were higher than those obtained using drop tensiometry because of longer duration of tensiometry measurement. The SICs of the LNCs were approximately 10-fold higher than the SICs of the binary and ternary mixtures of LNC ingredients or the CMC of MHS. The SIC depended on the mass mixing ratio of MHS/triglycerides but not on the presence of lecithin.

The surface active properties of the LNCs offer new possibilities as surfactants in various medical and pharmaceutical applications.
